# Identification of Priority Conservation Areas for Natural Heritage Sites Integrating Landscape Ecological Risks and Ecosystem Services: A Case Study in the Bogda, China

**DOI:** 10.3390/ijerph19042044

**Published:** 2022-02-11

**Authors:** Tian Wang, Xiaodong Chen, Xin Zheng, Yayan Lu, Fang Han, Zhaoping Yang

**Affiliations:** 1 State Key Laboratory of Desert and Oasis Ecology, Xinjiang Institute of Ecology and Geography, Chinese Academy of Sciences, Urumqi 830011, China; wangtian181@mails.ucas.ac.cn (T.W.); chenxiaodong181@mails.ucas.ac.cn (X.C.); luyayan16@mails.ucas.ac.cn (Y.L.); hanfang@ms.xjb.ac.cn (F.H.); 2 University of Chinese Academy of Sciences, Beijing 100049, China; zhengxin0722@igsnrr.ac.cn; 3 Institute of Geographic Sciences and Natural Resources Research, Chinese Academy of Sciences, Beijing 100101, China

**Keywords:** landscape ecological risk, ecosystem services, priority conservation areas, scenarios, natural heritage site

## Abstract

The conservation of World Natural Heritage Sites has become a global concern. The identification of priority conservation areas can preserve the value of heritage sites while promoting sustainable development, which is important for balancing the conservation and development of heritage sites. This paper proposes an integrated framework for the identification of priority conservation areas for natural heritage sites based on landscape ecological risks (LERs) and ecosystem services (ESs), taking the Bogda heritage site in Xinjiang, China as a case study. The innovative approach combined the natural and cultural elements of natural heritage sites and included the following steps: (1) the LER index, Integrated Valuation of Ecosystem Services and Tradeoffs (InVEST) model and questionnaire method were adopted to assess the LERs and ESs of Bogda heritage sites during 1990–2018; (2) ordered weighted averaging (OWA) was used to identify conservation priorities by weighing LERs and ESs; and (3) the optimal priority conservation area was determined by comparing the conservation efficiencies under different scenarios. The results revealed that the LER, carbon storage (CS), habitat quality (HQ), aesthetic value (AV), and recreational value (RV) showed significant spatiotemporal variation. The most suitable priority conservation area was located at the central forestlands and high-coverage grasslands, with conservation efficiencies of 1.16, 2.91, 1.96, 1.03, and 1.21 for LER, CS, HQ, AV, and RV, respectively. Our study demonstrated that integrating LERs and ESs is a comprehensive and effective approach to identifying conservation priorities for heritage sites. The results can provide decision support for the conservation of the Bogda heritage site and a methodological reference for identifying conservation priorities for natural heritage sites. Furthermore, this study is also an effective application of LERs and ESs in identifying priority conservation areas.

## 1. Introduction

World Natural Heritage Sites are the common heritage of humanity and have outstanding value regarding geology, bioecological processes, species habitats, and aesthetics [1]. However, irrational human activities have caused severe environmental disturbance and ecological degradation of heritage sites, such as landscape disruption and loss of biodiversity, among other negative effects [2,3]. The identification of conservation priorities is an essential and crucial step in the conservation of natural heritage sites [4]. Existing approaches to the delineation of priority conservation areas for heritage sites generally focus only on the conservation of natural values [5], and they are no longer adequate for the conservation of multiple values. Therefore, the delineation of priority conservation areas that can protect multiple values of heritage sites has become an urgent issue.

With the growing understanding of the conservation objectives of protected areas, from the initial focus on wildlife conservation, protected areas are now more concerned with the conservation of natural, socioeconomic objectives [6]. There have been studies to identify priority conservation areas in areas such as cities, watersheds, and mountains based on species diversity, ecosystem services, and socioeconomic costs [7,8,9], but there is a lack of studies to identify priority conservation areas in natural heritage sites. Most studies have adopted methods such as overlay analysis and multicriteria decision making, among others [8,10,11]. Ordered weighted averaging can effectively weigh multiple factors and has been repeatedly shown to be suitable for the identification of priority conservation areas [12,13].

Landscape ecological risk (LER) refers to the possible adverse consequences of the interaction of landscape patterns and ecological processes under the impact of natural or human factors [12,14,15]. Analyzing the response of landscape elements, patterns, and ecological processes to intrinsic risk sources and external disturbances, it is determined that regional landscape components, structures, and processes are affected by human activities or natural hazards [16,17]. LERs have been adopted to serve as a decision-making reference for regional risk prevention and promote the management of landscape patterns in mountainous, wetland, and coal mine areas [16,18,19,20]. Nevertheless, fewer studies have focused on how to link LERs to the identification of priority conservation areas for heritage sites. Ecosystem services (ESs) are the benefits that humankind can derive from ecosystems, which can bridge ecosystems and human well-being [21,22]. ESs are classified into four categories: provisioning services, regulating services, supporting services, and cultural services [23]. With the growing interest in the spiritual benefits provided by ecosystems, more attention should be given to cultural ecosystem services, especially for heritage sites [24,25]. ESs of heritage sites are an important object of conservation because they embody the outstanding contribution of heritage sites to the enhancement of human well-being [26]. The Integrated Valuation of Ecosystem Services and Tradeoffs (InVEST) model is a relatively mature and widely used ES assessment model [27,28]. On the one hand, LERs indicate the degree of risk to the landscape and can provide guidance for the reduction of ecological risk. On the other hand, ESs are an important bridge between ecosystems and human well-being and should be considered as an important factor for conservation. Hence, how to integrate LERs and ESs into the identification of priority conservation areas for natural heritage sites is still challenging.

The Bogda heritage site was selected as a case study. This site is located in Xinjiang, China and was designated as a World Natural Heritage Site in 2013. The aim of this study is to propose and empirically demonstrate a method for identifying priority conservation areas for natural heritage sites based on LERs and ESs. Specifically, our main research aims are: (1) to analyze the spatiotemporal changes in LERs and ESs of the heritage site during 1990–2018; (2) to propose a framework for identifying conservation priorities for heritage sites that combines LERs and ESs and generates multiple conservation priority scenarios; and (3) to determine the optimal conservation priority scenarios based on conservation efficiency comparisons. The study results are expected to enrich research on the identification of priority conservation areas for natural heritage sites and provide a reference for managers to implement conservation measures for heritage sites.

## 2. Materials and Methods

### 2.1. Study Framework

**Figure 1:**
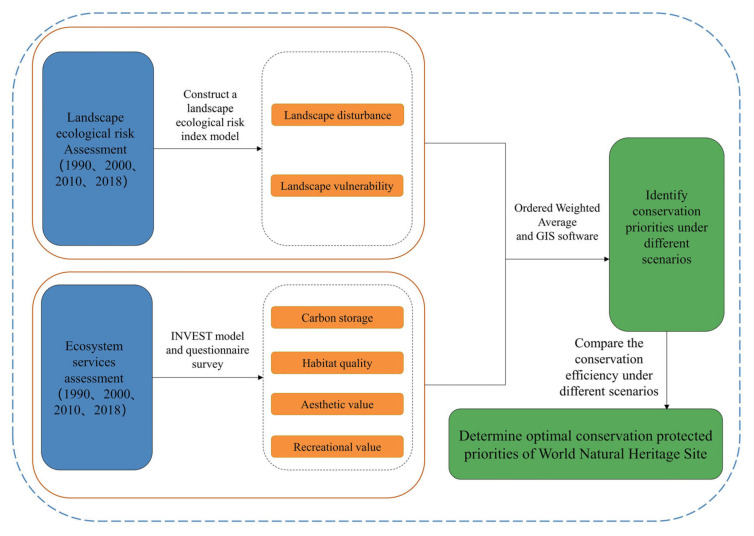
Study framework to integrate LERs and ESs to identify priority conservation areas.

We first assessed the LERs and ESs of the Bogda heritage site in 1990, 2000, 2010, and 2018. Then, we combined LERs and ESs to determine the priority conservation areas under different scenarios and compared the protection efficiencies to determine the most suitable protection area. The details are described below (Figure 1).

### 2.2. Study Area

**Figure 2:**
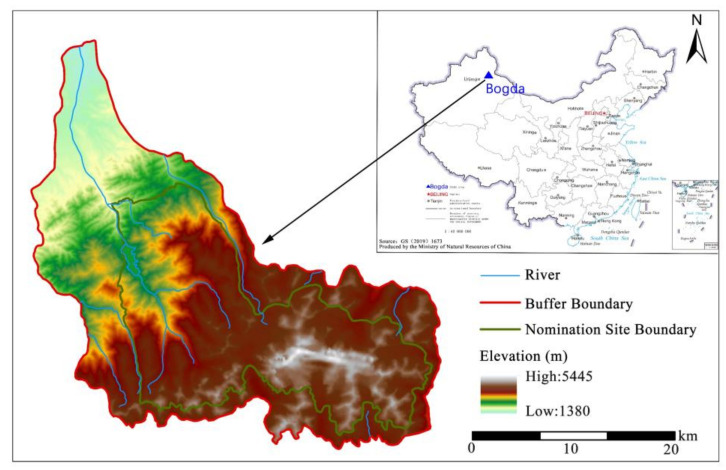
Location of Bogda heritage site, China.

Bogda is located at the eastern part of the Tianshan Mountains in Xinjiang, China (Figure 2). The elevation ranges from 1340 m to 5445 m, with a vertical elevation difference of nearly 4065 m. The climate is classified as continental temperate climate. The annual average temperature is 2.55 °C, and the annual average precipitation is 443.9 mm.

It is the most typical representative of the mountain altitudinal belts on the northern slope of the Tianshan Mountains in Xinjiang. The unique and diverse habitats support many species, including 127 animal species listed on the IUCN Red List of Species. According to the Chinese vegetation classification, there are eight vegetation types, eighteen vegetation subtypes, and twenty-seven formations. With various natural landscapes, including mountains, lakes, glaciers, forestlands, and grasslands, it presents abundant landscape diversity and high aesthetic value. Bogda is one of the components of the Xinjiang Tianshan heritage series, which was added to the World Natural Heritage List in 2013 for its outstanding value of bioecological processes and landscape aesthetics [1]. Currently, some adverse factors endanger the ecological environment and cultural values of heritage sites, and there is a need to scientifically identify protection priority areas.

### 2.3. Data Sources

The land-use and land-cover (LULC) datasets in Bogda for 1990, 2000, 2010, and 2018 were obtained at the Data Center for Resources and Environmental Sciences, Chinese Academy of Sciences (RESDC) (http://www.resdc.cn, 30 June 2021) with good quality control, which included 6 primary types (i.e., arable land, forestland, grassland, water, construction land, and unused land) and 25 secondary types. The road data for 1990, 2000, 2010, and 2018 were also obtained from the RESDC. The vector data for boundaries, grazing sites, and tourism services were derived from the Xinjiang Tianshan World Natural Heritage Site declaration. These panoramic photos were acquired by the research team in the field, and the questionnaire data were acquired from field surveys.

### 2.4. Assessment of Landscape Ecological Risk

#### 2.4.1. Division of Assessment Units

Based on the sample area requirements of landscape ecology [29], we divided the Bogda heritage site into 243 assessment units by a grid of 2 km × 2 km. Then, the LER index of each unit was calculated and assigned to the centroid of each cell as an attribute value, and the LERs of the whole study area were obtained through spatial interpolation.

#### 2.4.2. Construction of Landscape Ecological Risk Index

LER assessment provides a new perspective for the study of landscape pattern–ecological process feedbacks, which can effectively support ecosystem management. The magnitude of LER depends on the strength of the regional ecosystem affected by external disturbances and the magnitude of internal resistance [30]. This study selected the landscape disturbance index and vulnerability index to construct the LER index [31,32] and analyzed the spatiotemporal changes in LERs.

(1) Landscape disturbance index

The landscape disturbance index reflects the disturbance degree to the ecosystem represented by different landscapes. The higher the disturbance in this region, the higher the landscape disturbance index. The landscape disturbance index can be calculated as follows:

(1) $$E_{i}=aC_{i}+bN_{i}+cD_{i},$$

where *E_i_* is the landscape disturbance index of landscape type *i*, *C_i_*, *N_i_*, and *D_i_* are the landscape fragmentation degree, landscape separation degree, and landscape fractal dimension, respectively; *a*, *b*, and *c* represent the weights of the three landscape indexes, and we used the expert scoring method to obtain the weights as 0.5, 0.3, and 0.2, respectively.

(2) Landscape vulnerability index

The landscape vulnerability index indicates the vulnerability of the internal structure of the ecosystem represented by different landscapes, which reflects the resistance of different landscapes to external disturbances. The Bogda landscape types were ranked from lowest to highest vulnerability according to expert ratings [16,32]: lake, bare rocky texture, permanent glaciers and snow, dryland, high-coverage grassland, medium-coverage grassland, low-coverage grassland, forestland, shrubland, and sparse woodland. The landscape vulnerability index (*F_i_*) was calculated by the Z-score standardization method.

(3) Landscape ecological risk index

LERs were obtained by integrating the landscape index and regional ecological risk based on the assessment unit of the study area. The equation is as follows:

(2) $$LER_{k}=\sum_{i=1}^{n}\frac{A_{i}}{A}\left(E_{i}\times F_{i}\right),$$

where *LER_k_* is the landscape ecological risk in assessment unit *k*; *A_i_* is the area of land cover type *i* in evaluation unit *k*; *A* is the area of evaluation unit *k*; and *E_i_* and *F_i_* are the landscape disturbance index value and the vulnerability index value of land cover type *i* in assessment unit *k*, respectively. The risk values of the 243 units were calculated and assigned to the centroid of each unit separately as attribute values. Then, spatial interpolation was used to obtain the LERs of Bogda. The LERs were generally classified into five levels to distinguish different risk levels [17,32] and thus developed different management strategies.

### 2.5. Quantification of Ecosystem Services

ESs are divided into four categories: provisioning services, regulating services, cultural services, and supporting services [23]. Considering the outstanding contribution of the Bogda heritage site to climate regulation, habitat provision, and cultural values [1,5], the study selected four ecosystem services for quantitative assessment: carbon storage (CS), habitat quality (HQ), aesthetic value (AV), and recreational value (RV). The InVEST model was adopted to quantify CS and HQ, and perception-based questionnaire surveys were used to assess AV and RV.

#### 2.5.1. Carbon Storage

The CS module in the InVEST model requires LULC data and carbon pool data (aboveground biological carbon density, belowground biological carbon density, dead organic matter carbon density, and soil carbon density). The carbon density table for Bogda was obtained by referring to the existing literature [33,34,35]. The LULC data of Bogda for 1990, 2000, 2010, and 2018 and the carbon density tables were input into the carbon storage module in the InVEST model to obtain the CS map of Bogda.

#### 2.5.2. Habitat Quality

HQ is the capacity of the ecosystem to supply essential goods and services to individuals and groups [36,37]. The HQ model of InVEST obtains HQ maps by analyzing LULC maps and their calculation of the threat level to biodiversity. With reference to existing studies and the actual situation in the study area [38,39], roads, grazing sites, and tourist facilities were selected as habitat threat factors, and the maximum threat distance and factor weights were determined by expert scoring. The LULC maps, threat source data, and related parameters were put into the HQ module to obtain a spatial distribution map of habitat quality in Bogda. More operational details can be found in the InVEST User’s Guide. The habitat quality of Bogda was classified into three levels with the Jenks methods of natural breaks [37,39]: low habitat quality (0–0.2), medium habitat quality (0.2–0.8), and high habitat quality (0.8–1).

#### 2.5.3. Aesthetic Value and Recreational Value

We surveyed public perceptions of cultural ecosystem services in Bogda, and the two most perceived values were AV and RV. Therefore, this study elected to assess AV and RV. With aesthetic appreciation as an anthropocentric phenomenon, landscape aesthetics can be understood as the human perception and enjoyment of a particular place [25]. Scholars have conducted many studies on aesthetic value assessment, and the main research methods include indicator systems, machine learning, and questionnaire surveys [40,41,42]. In this paper, the perception-based questionnaire was selected to assess the AV of Bogda. Respondents included experts, managers, and tourists, and there were 380 valid questionnaires. The choices of different respondents were averaged to derive quantitative indicators of aesthetic value. According to the inscription document of Xinjiang Tianshan, we identified twenty-one resource spots with typical value representation to be selected as sample sites. Standardized photographic questionnaires were used, and photographic panoramas depicting the most representative landscapes were created for all 21 sample sites. Respondents were first shown a preview of all the photos, then were shown them again in a casual manner and were asked to rate each location on a scale of 1 to 10 based on purely aesthetic criteria. We calculated the average score of 380 questionnaires to obtain the AV of each sample point and then obtained the AV map by inverse distance weighted (IDW) interpolation in GIS [43].

RV refers to the direct or indirect benefits generated when tourists engage in leisure activities, such as scenery viewing, picnics, and other tourism activities [44,45,46]. RV is subjective and intangible and difficult to assess without human participation [45]. The assessment of AV used a questionnaire survey based on visitor perceptions, with 380 valid questionnaires. Twenty-one recreation sample sites were identified, and visitors scored the RV of sample sites in the range of 1–10 according to their perceptions. The mean value of the questionnaires was calculated to obtain the recreation value score of each sample site, and finally, an AV map was obtained by IDW interpolation [43].

### 2.6. Selection of Optimal Conservation Priorities

#### 2.6.1. OWA Algorithm

The OWA algorithm can balance multiple elements to identify conservation priorities [47,48]. This algorithm was first proposed in 1988 [49], and the equation is as follows.

(3) $$OWA_{\left(a_{xj}\right)}=\sum_{x}^{n}\omega_{x}S_{xj},\;\;\;\;\;\;(\omega_{x}\in \left[0,1\right],\;\sum_{x}^{n}\omega_{x}=1,\;\mathrm{for}\;x\;\mathrm{and}\;j=1,2,3\ldots ,n),$$

(4) $$S_{xj}=\frac{S-S_{min}}{S_{max}-S_{min}},$$

where $a_{xj}$ is the normalized five raster criterion layers (LER, CS, HQ, AV, and RV) with the normalization method shown in Equation (4). *S_xj_* is the new raster layer obtained after sorting from largest to smallest with the five raster layers $a_{xj}$. $\omega_{x}$ is the new ordered weight of the new dataset $S_{xj}$. x is the number of raster criterion layers.

The following equations can calculate the risks and tradeoffs corresponding to different ordered weights:

(5) $$risk=\frac{\sum_{x}^{n}\left(n-i\right)\omega_{x}}{n-1}\;\;\;\;\;\;\;\left(0\leq risk\leq 1\right),$$

(6) $$tradeoff=1-\sqrt{\frac{n\sum_{x}^{n}{\left(\omega_{x}-\frac{1}{n}\right)}^{2}}{n-1}}\;\;\;\;\;\;\;\;\;\left(0\leq tradeoff\leq 1\right),$$

where x is the number of raster criterion layers. $\omega_{x}$ is the new ordered weight of raster criterion layers. Countless scenarios will be generated by changing the risks and tradeoffs. If decision-makers choose the higher risk, they will assign high weights to the higher raster indicator layers and vice versa. If they want to obtain the highest tradeoff, they must assign the same weight to the raster criterion layers. If they assign the highest weight, one, to the highest or lowest raster layer, they will obtain the lowest tradeoff, zero. The higher the tradeoff, the more even the weight.

According to the definition of risk and tradeoff, the maximum tradeoff under the same risk is solved to set the combination of different weights.

(7) $$maxtradeoff=1-\sqrt{\frac{n\sum_{x}^{n}{\left(\omega_{x}-\frac{1}{n}\right)}^{2}}{n-1}}\;\;\;\;\;\;\;\;\;\left(0\leq tradeoff\leq 1\right),$$

(8) $$\overset{n}{\underset{x}{\sum }}\omega_{x}=1,\;x=1,2,3\ldots ,n,$$

(9) $$\omega_{x}\in \left[0,1\right],$$

This study set the risk to 0–1 with an interval of 0.1 to save computational time and difficulty [12,13], and 11 scenarios were generated.

#### 2.6.2. Selection of the Optimal Conservation Priorities

The conservation priorities were obtained by extracting 20% of the OWA grid map with the largest grid values [12,13]. Different scenarios generated different priorities with different conservation efficiencies. By comparing different conservation efficiencies, the optimal scenario corresponding to the best conservation efficiency was determined. The conservation efficiency was calculated as follows.

(10) $$E=\frac{\bar{ES_{C}}}{\bar{ES_{O}}},$$

*E* is the conservation efficiency of a specific indicator in the conservation priorities, *ES_C_* is the average value of the specific indicator in the conservation priorities, and *ES_O_* is the average value of a specific indicator in the entire study area.

## 3. Results

### 3.1. Spatiotemporal Changes of Landscape Ecological Risk

**Figure 3:**
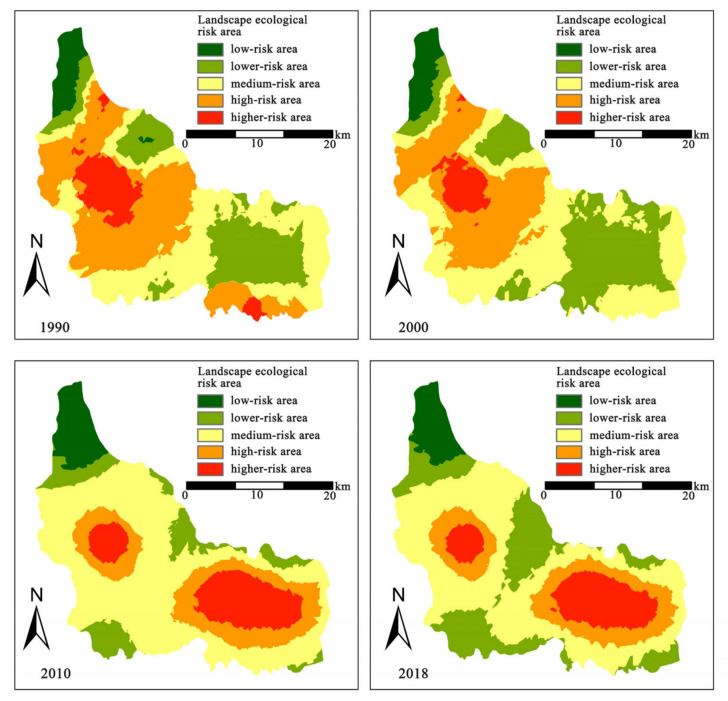
Spatial distribution and changes of LER in Bogda from 1990 to 2018.

According to the classification of the LER index results, the Bogda heritage site was classified into five classes (Figure 3): low-risk area (<0.026), lower-risk area (0.026–0.033), medium-risk area (0.033–0.038), higher-risk area (0.038–0.045), and high-risk area (>0.045). Spatial differences are evident, with the high-risk areas mainly located in the central region and southeast of Bogda peak, while the medium- and low-risk areas were distributed around the high-risk areas. From a temporal perspective, LER generally decreased from 1990 to 2018, and the average risk value in the study area increased slightly in 2010. By comparing the change in risk levels over the four phases, it is clear that the area of higher-risk zones decreased from 35% to 16%, the area of high-risk zones increased from 4% to 14%, the area of medium-risk zones decreased from 34% to 43%, and the low- and lower-risk zones were floating up and down with no significant change in proportion.

### 3.2. Spatiotemporal Changes of Ecosystem Services

#### 3.2.1. Carbon Storage

**Figure 4:**
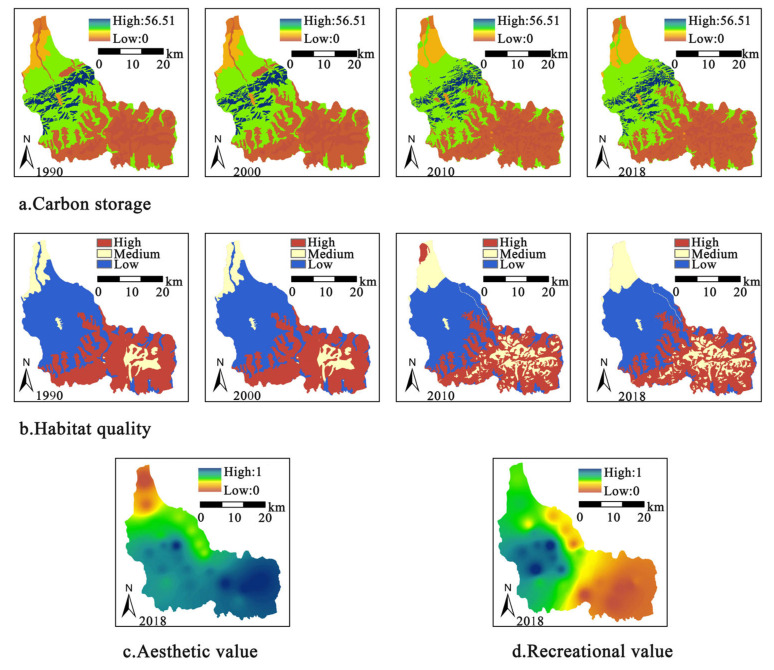
Spatial distribution and changes of CS (**a**), HQ (**b**), AV (**c**), and RV (**d**) in Bogda.

There were significant differences in the spatial patterns of CS in Bogda. The high-value areas were mainly distributed in the central forestland, while the low-value areas were mainly in the permanent glacier and snow area and bare rock texture in the southeast (Figure 4a). Temporally, the CS showed a slight change of first decreasing and then increasing. In 1990, the CS in Bogda was 10.67 × 10^6^ t, and in 2010, it was 10.46 × 10^6^ t, with a small decrease of 0.19 × 10^6^ t. In 2018, the CS increased to 10.58 × 10^6^ t.

#### 3.2.2. Habitat Quality

The results found significant spatial variation in HQ in Bogda (Figure 4b). The western areas of the heritage site had better habitat quality than the eastern areas. Areas of high habitat quality are mainly found in woodlands and high cover grasslands, while areas of low HQ are found in permanent glaciers and snow and bare rock textures. The overall HQ of the heritage site showed a modest change between 1990 and 2018, with a small downwards trend in the average value of HQ. By comparing the area of each level zone of HQ across four phases, the area of low HQ areas decreased from 43% to 39% and the area of high HQ areas decreased from 47% to 43%; however, the area of medium HQ areas increased from 11% to 18%.

#### 3.2.3. Aesthetic Value and Recreation Value

The results revealed that AV was characterized by spatial differentiation in 2018 (Figure 4c). High AV areas were mainly located in the southeastern Bogda peak and the central Tianchi Lake region, while low AV areas were distributed in the northwestern grazing sites. Combined with the LUCC map, it was found that the AVs of forestlands, lakes, and glaciers were relatively high, while the AVs of drylands and low-coverage grasslands were low. The natural vertical zone of Bogda forestland was the mountainous evergreen coniferous forest zone, the main component of which was the snowy ridge spruce group system with high appreciation value.

The high RV areas were concentrated in the central part of the Bogda heritage site, where Tianchi Lake and Maya Mountain were located, and there was a similarity between those found and AVs (Figure 4d). The areas with low RVs were distributed in the southeastern Bogda peak region. Overall, the RV in the western region was higher than that in the eastern region. Therefore, the spatial distributions of RV and AV have a certain similarity.

### 3.3. Conservation Priorities under Different Scenarios

According to Equations (6)–(10), the weights and tradeoffs for each scenario were obtained. The 5 indicator layers were multiplied with the corresponding weights to obtain the raster layers for 11 scenarios (Table 1).

**Figure 5:**
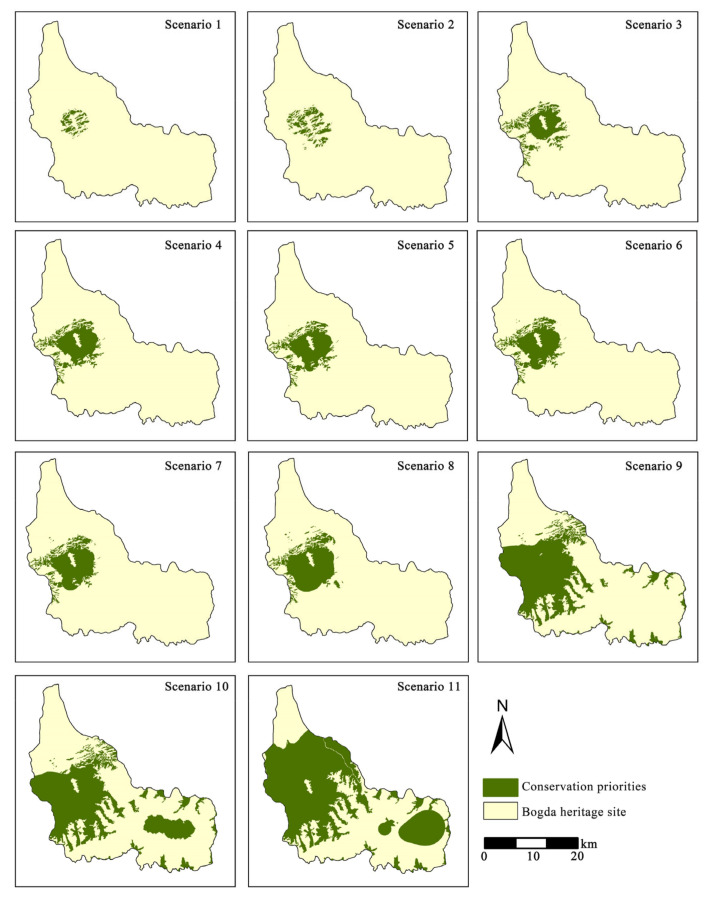
Conservation priorities under different scenarios.

**Figure 6:**
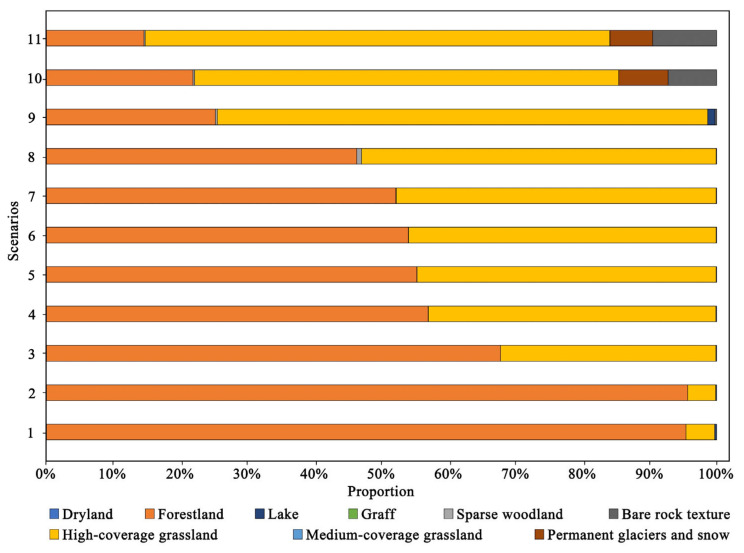
Area percentage of different LULC types under 11 scenarios.

The location of the priority conservation areas changed as the risk value increased (Figure 5). Generally, the priority conservation areas were all concentrated in the central region of the Bogda heritage site, mainly located in Tianchi Lake and its surrounding forestland. Scenarios 1 and 11 were two scenarios with more extreme decision-making preferences, which would result in an extreme value of protected areas and would deviate significantly from reality. Hence, these two scenarios were not considered in this study. Under scenarios 2–8, the range of conservation priorities gradually broadened to the surrounding areas. Scenarios 9–10 showed that the conservation areas became more dispersed with spreading to the southeast. Among the 11 scenarios, forestland and high-coverage grassland in the conservation priorities accounted for more than 96% of the total area, with forestland accounting for more than 53%, which also suggested that forestland plays a vital role in ESs (Figure 6), so conservation areas were more concentrated in forestlands.

### 3.4. Conservation Efficiency under Different Scenarios and the Optimal Priority Conservation Area

Under the 11 conservation scenarios, LERs and ESs were higher than the regional average, apart from CS, AV, and RV for scenario 2 and LER for scenario 4. The LER and CS were most efficiently conserved in scenario 3, with 1.16 and 2.91, respectively. The highest conservation efficiency of HQ was 1.96 for scenarios 3–8, and the highest conservation efficiencies of AV and RV were 1.04 and 1.22 for scenarios 5, 6, and 7, respectively. Scenario 3 has the highest conservation efficiency of 1.65 on average, so it was selected as the best scenario, and the corresponding protected area in scenario 3 is the optimal conservation priority of Bogda (Table 2).

In scenario 3, the conservation efficiencies of LER, CS, HQ, AV, and RV were 1.16, 2.91, 1.96, 1.03, and 1.21, respectively, with CS being the most efficient. Forestlands accounted for the most significant proportion of the conservation priorities, with 67.75% in scenario 3 (Figure 6), since they play an essential role in climate regulation and habitat provision, followed by high-coverage grasslands and lake.

## 4. Discussion

### 4.1. Spatiotemporal Changes of Landscape Ecological Risk

Our results showed that the high-value areas of LER in Bogda were distributed in the central Tianchi Lake region and southeastern Bogda alpine peaks. The central Tianchi region is concentrated on alpine lakes, mountain evergreen coniferous forests, mountain meadows, and grasslands [5]. The Tianshan Tianchi Scenic Spot is a national AAAA scenic spot, and overly frequent tourism activities interfere with regional ecological processes and threaten the ecological landscape of the heritage site [50]. Thus, it is necessary to achieve harmony between environmental protection and the development of heritage sites. The alpine peaks southeast of Bogda were the high-value area, where permanent glaciers and snow are predominantly located. Glaciers are very sensitive to climate change, and climate change has caused a continued and accelerated retreat of Bogda glaciers in recent years [51]. With the accelerating rate of climate change, the LER in the Bogda alpine zone will likely continue to increase in the future.

We inferred that the successful declaration of Bogda as a World Natural Heritage Site has had an impact on the landscape ecology. Bogda became a natural heritage site in 2013 as part of the Tianshan natural heritage sites series. National and local governments have given high priority to the protection of the ecological environment and resources of nominated heritage sites. A series of measures has gradually been implemented, such as the removal of destructive structures, the development of heritage protection regulations, and the implementation of ecological restoration projects. In addition, it is worth noticing that the reputation of the World Natural Heritage Site has promoted the rapid development of tourism. From 2010 to 2016, the number of visitors to scenic spots increased from 136 million to 210 million, and the increased intensity of tourism activities put enormous pressure on the ecological environment of heritage sites. Future research needs to pay more attention to determining the reasonable capacity of tourism in heritage sites and exploring more sustainable tourism development models for natural heritage sites.

### 4.2. Spatiotemporal Changes of Ecosystem Services

The high-value area of CS was mainly concentrated in the central region with high vegetation coverage, where mountain coniferous forest zones with altitudes ranging from 1650 m to 2750 m were mainly distributed. This finding also suggested that the forest ecosystem, as the largest terrestrial ecosystem carbon pool, contributes to reducing carbon emissions and maintaining climate stability [52,53]. The low-value area was distributed in the southeastern snow and glacier belt, with altitudes ranging from 3700 m to 5445 m and low vegetation coverage. We inferred that altitude and vegetation type may influence regional carbon storage. Elevation controls the gradient changes of local temperature and precipitation, which directly or indirectly affect the carbon density. Vegetation type and coverage may affect the capacity of regional carbon storage and cycling.

We found that the spatial pattern of HQ was similar to that of LULC, which was consistent with the results of Yang [37] and Yohannes et al. [54]; both found that HQ and LULC had similar spatial patterns. The high-value areas of HQ were mainly distributed in forestland and high-coverage grassland, where high vegetation coverage can effectively reduce threats from the external environment and provide a good survival environment for species. The low-value areas were located at the southeastern alpine glaciers and the northwestern drylands. Alpine glaciers are not suitable for species survival due to long-term low temperatures. The northwestern region contained many facilities, such as grazing sites, ethnic parks, and settlements, and anthropogenic disturbances caused the degradation of HQ. Therefore, the HQ in the areas disturbed by human activities was lower than that of other areas with relatively intact ecosystems.

The change in HQ over time reflected the fact that land use change has a significant impact on HQ. From 2000 to 2010, the area of forestland decreased by 9.19 km^2^ and the area converted from high- to medium-cover grassland was approximately 3.96 km^2^. At the same time, HQ decreased from 0.52 to 0.49, with a significant 4% decrease in the area of high-value HQ. From 2010 to 2018, the change in LULC was slight, but HQ improved overall. We inferred that development policies and management practices have contributed to the improvement in HQ. In 2010, the Management Committee of Tianshan Tianchi Scenic Area in Xinjiang demolished 46 structures covering an area of approximately 5.7 km^2^ within the Tianshan Tianchi Scenic Area jurisdiction and implemented a simultaneous grazing ban protection project. In 2013, Bogda was added to the UNESCO World Natural Heritage List. Since 2016, China has introduced a series of policies to promote ecological civilization and realize the green development concept that “clear mountains and clean water are valuable assets”. To protect the authenticity and integrity of the natural heritage site, Bogda has implemented strict conservation management measures, which have had a positive impact on HQ.

The results indicated that the AV score of Bogda was generally high, reflecting the outstanding aesthetic value of Bogda’s heritage site, which is one of the typical representatives of the alpine lake landscape in the Tianshan Mountains of Xinjiang [55]. To some extent, this result validated the previous finding that people prefer natural environments to urban environments in terms of aesthetics, especially mountainous environments [56,57]. The areas of high AV in Bogda were distributed in the southeastern Bogda peak, and a previous study found that mountain tops and forested water bodies have higher AV [58], which was consistent with our findings. The areas with the second highest AV were the coniferous forestland and alpine meadow areas in the center. The areas with lower AV were drylands, low-coverage grasslands, and sparse woodlands in the northwest, where the AV of the landscape decreased with decreasing vegetation [59,60].

The high-value area of RV was mainly distributed in the central Tianchi area, which was similar to the spatial distribution of AV. However, the RV of the Bogda peak with high AV was relatively low. When evaluating RV, visitors generally considered recreational suitability, such as climate comfort and accessibility, in addition to landscape beauty. The central Tianchi Lake area is the core area of the Tianshan Tianchi Scenic Area, where the landscape and tourist facilities of alpine lakes and snowy mountain spruce with high AV are distributed. In the northwest of the Bogda, tourist attractions such as the Ethnic Park have been built based on the original settlements, where visitors can visit and experience. The AV of Bogda Peak was high, but the conditions of low temperature and high altitude reduced the suitability for recreation. Meanwhile, only adventure activities and scientific research have been carried out, currently. Hence, the perceived visitor RV in this region was relatively low.

### 4.3. Identification of Conservation Priorities for World Natural Heritage Sites

It is necessary to integrate natural and cultural factors in the identification of conservation priorities for heritage sites. Since the purpose of protected areas was primarily to protect the ecological environment, the extraction of protected areas mainly considered only natural elements [6,8]. However, this method would not apply to the identification of conservation priorities for natural heritage sites. World heritage is not only a rare and irreplaceable treasure of humanity recognized by UNESCO but also a heritage site and natural landscape recognized by all mankind as having outstanding significance and universal value [1]. Bogda was added as a World Natural Heritage Site for its typical bioecological processes and aesthetic value. The heritage site is an outstanding example of the succession of biomes in the mountain ecosystems of the arid zone and an area of outstanding natural beauty and aesthetic importance. It hosts more than 3 million visitors annually and provides tourists with multiple cultural ecosystem services, such as recreation, aesthetics, and scientific education. Therefore, it is necessary to consider both natural and cultural factors in identifying priority conservation areas of natural heritage sites and especially not to neglect the cultural benefits. When assessing AV and RV, this study adopted a perception-based evaluation method to maximize public participation in identifying conservation priorities for a “win–win” balance between human well-being and ecological conservation. In this study, five indicator layers were comprehensively considered when identifying conservation priorities for heritage sites, of which two were cultural elements. If only three natural factors (LER, CS, and HQ) were considered, the results varied greatly (Table 1). Moreover, none of the 11 scenarios can simultaneously guarantee the maximum conservation efficiency of all elements. We can only find a tradeoff scenario with the relatively highest conservation efficiency. Therefore, it is essential to identify conservation priorities for heritage sites by considering natural and cultural factors integrally.

Compared with the original delineation of core and buffer zones of natural heritage sites, the conservation priorities extracted in this study based on an integrated consideration of LER and ESs are more detailed and specific, which is the main reason for the differences between them. It is important to emphasize that this study attempted to balance the conservation of the landscape ecology and ESs of the heritage site and provided a reference for the identification of priority conservation areas for the heritage site.

### 4.4. Conservation Priorities under Different Scenarios

The proportion of LULC types and the composition of priority conservation areas differed under different scenarios. Forestlands (53%) were the most represented land-use type in the priority protected areas, followed by high-coverage grassland (43%). Cultural ecosystem services were considered in the extraction of priority conservation areas for heritage sites so that the final extracted conservation priorities were more concentrated in forestlands and high-coverage grasslands. The same result was found in the extraction of priority protected areas in the Guanzhong-Tianshui Economic Region [12].

None of the scenarios can guarantee the highest conservation efficiency for LER and all ESs at once, which implies that policymakers are responsible for assigning weights to different decision indicator layers to achieve tradeoffs between multiple elements when dividing protected areas. Under scenario 3, the highest conservation efficiency occurred in CS and HQ. Bogda is a typical natural heritage site of the mountain ecological process type, and the conservation of CS and HQ is crucial for the heritage site. Hence, this optimal conservation scenario was applied to the Bogda natural heritage site.

## 5. Conclusions

This study proposed a methodology for integrating LERs and ESs (CS, HQ, AV, and RV) to identify priority conservation areas at the Bogda natural heritage site. Both natural and cultural factors were considered to ensure that the identified priority conservation areas would protect the natural environment while preserving the cultural benefits. A total of 11 scenarios were considered to identify the best protected areas through the OWA method. Although each protected area gained good conservation efficiency for several factors, scenario 3 proved to be the best scenario with conservation efficiencies for LER, CS, HQ, AV, and RV of 1.16, 2.91, 1.96, 1.03, and 1.21, respectively. This study provided a reference for identifying priority conservation areas for natural heritage sites.

## Figures and Tables

**Table 1:** Tradeoff and weight of 5 indicator layers under 11 scenarios.

Scenario	ω1	ω2	ω3	ω4	ω5	**Risk**	**Tradeoff**
1	0	0	0	0	1	0	0
2	0	0	0.03	0.33	0.63	0.10	0.37
3	0	0.04	0.18	0.32	0.46	0.20	0.57
4	0.04	0.12	0.2	0.28	0.36	0.30	0.71
5	0.12	0.16	0.2	0.24	0.28	0.40	0.86
6	0.2	0.2	0.2	0.2	0.2	0.50	1
7	0.28	0.24	0.2	0.16	0.12	0.60	0.86
8	0.36	0.28	0.2	0.12	0.04	0.70	0.71
9	0.46	0.32	0.18	0.04	0	0.80	0.57
10	0.63	0.33	0.03	0	0	0.90	0.37
11	1	0	0	0	0	1	0

**Table 2:** Protection efficiency under different scenarios.

Scenario	LER	Carbon Storage	Habitat Quality	Aesthetic	Recreation	Average
1	1.28	3.43	1.95	1.04	1.22	1.78
2	1.13	0.85	1.06	0.93	0.99	0.99
3	1.16	2.91	1.96	1.03	1.21	1.65
4	0.95	1.74	1.96	0.98	1.10	1.35
5	1.14	2.68	1.96	1.04	1.22	1.61
6	1.14	2.66	1.96	1.04	1.22	1.60
7	1.13	2.62	1.96	1.04	1.22	1.59
8	1.10	2.50	1.96	1.03	1.21	1.56
9	1.02	2.11	1.95	1.02	1.14	1.45
10	1.07	1.83	1.71	1.03	1.08	1.35
11	1.03	1.68	1.68	1.00	1.05	1.29

## Data Availability

The dataset is provided by Data Center for Resources and Environmental Sciences, Chinese Academy of Sciences (RESDC) (http://www.resdc.cn, accessed on 30 June 2021).
